# Supplementary material for: Optimization of Precursor Synthesis Conditions of (2S,4S)4–[18F]FPArg and Its Application in Glioma Imaging
Source: Pharmaceuticals (Basel). 2022 Jul 29;15(8):946. doi: 10.3390/ph15080946 (PMC9416586; doi:10.3390/ph15080946)

Supplemental Information

Optimization of Precursor Synthesis Conditions of (2S,4S)4–[^{18}F]FPArg and Its Application in Glioma Imaging

**Yong Huang [1], Lu Zhang [2], Meng Wang [3], Chengze Li [1], Wei Zheng [2], Hualong Chen [2], Ying Liang [1,*]
and Zehui Wu [2,*]**

[1] Department of Nuclear Medicine, National Cancer Center, National Clinical Research Center for Cancer, Cancer Hospital & Shenzhen Hospital, Chinese Academy of Medical Sciences and Peking Union Medical College, Shenzhen 518116, China; 13260455651@163.com (Y.H.); lichengze1915@163.com (C.L.)

[2] Beijing Institute of Brain Disorders, Laboratory of Brain Disorders, Ministry of Science and Technology, Collaborative Innovation Center for Brain Disorders, Capital Medical University, Beijing 100069, China; zhanglu08292021@163.com (L.Z.); zhengwei2019@ccmu.edu.cn (W.Z.); chenhl@ccmu.edu.cn (H.C.)

[3] GDMPA Key Laboratory for Quality Control and Evaluation of Radiopharmaceuticals, Department of
Nuclear Medicine, Nanfang Hospital, Southern Medical University, Guangzhou 510515, China; 64878893@163.com

* Correspondence: liangy_2000@sina.com (Y.L.); wzhhuey2012@ccmu.edu.cn (Z.W.)

1. Radiolabeling and Efflux Experiments 2
2. MicroPET-CT imaging. 3
3. Estimated human dosimetry of (2S,4S)4-[^{18}F]FPArg 6
4. NMR of compound **4**. 7

1. Radiolabeling and Efflux Experiments

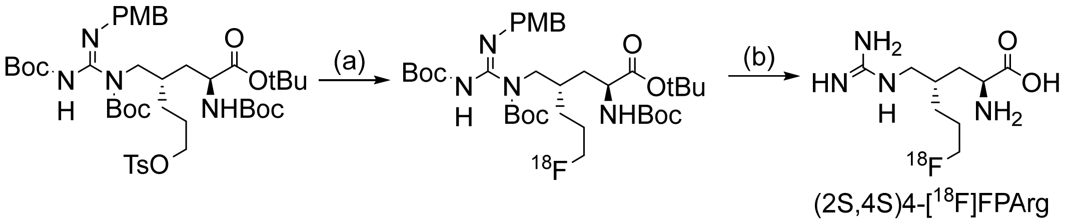

Scheme S1. Radiosynthesis of (2S,4S)4-[^{18}F]FPArg, Reagents and conditions: (a) 1 mL of 18-crown-6/$KHCO_3$ (320 mg of 18-crown-6 in 18.6 mL of ACN/58 mg of $KHCO_3$ in 3.4 mL of water), 100 °C, 15 min, tert-amyl alcohol and acetonitrile (9/1); (b) TFA, anisole, 60 °C, 5 min.

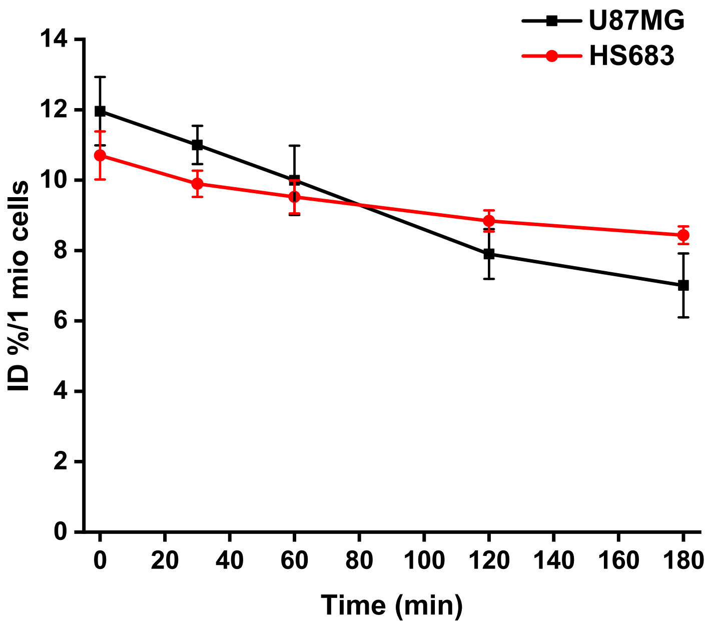

Figure S1. Efflux kinetics of (2S,4S)4-[^{18}F]FPArg after incubation of U87MG and HS683-Luc cells with radiolabeled compounds for 60 min followed by incubation with a compound-free medium for 0-180 min.

2. MicroPET-CT imaging.

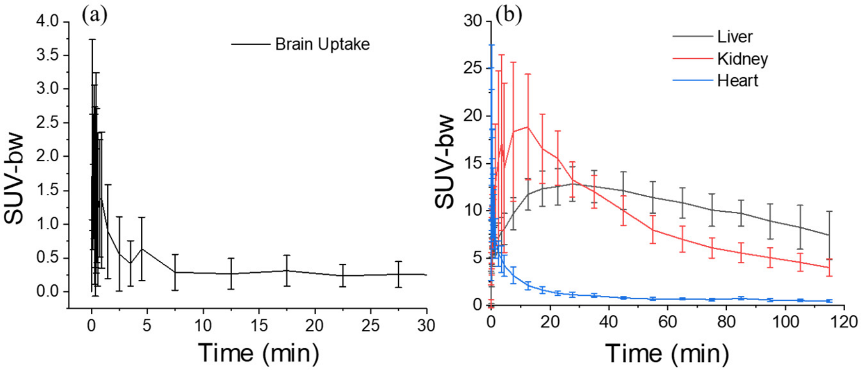

Figure S2. Time-activity curves of (2S,4S)4-[^{18}F]FPArg uptake in U87MG tumor-bearing nude mice brain (a), liver, kidney and heart (b).

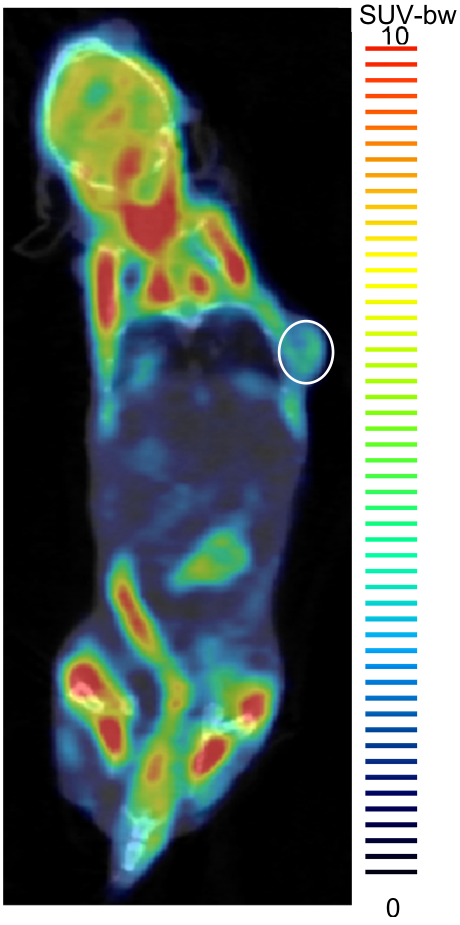

Figure S3. The microPET-CT image of [^{18}F]FDG in U87MG tumor-bearing nude mice, where white circle indicates glioma area.

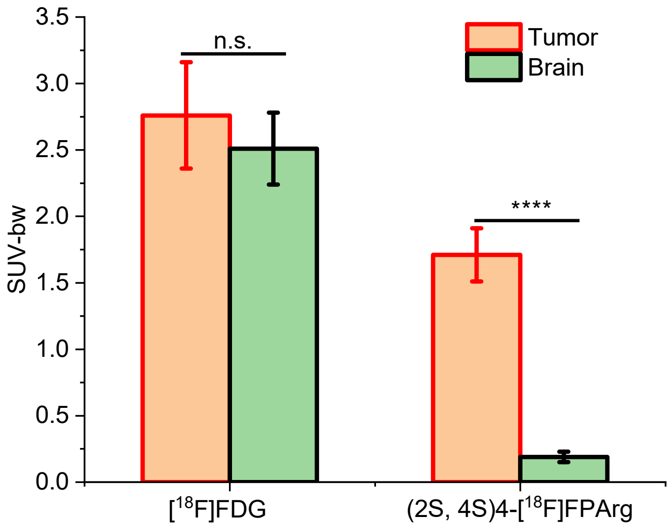

Figure S4. The SUV of [^{18}F]FDG and (2S, 4S)4-[^{18}F]FPArg uptake in brain and tumor of nude mice bearing HS683-Luc tumor at 60 min point (n.s.= no significance, ****, $p < 0.0001$).

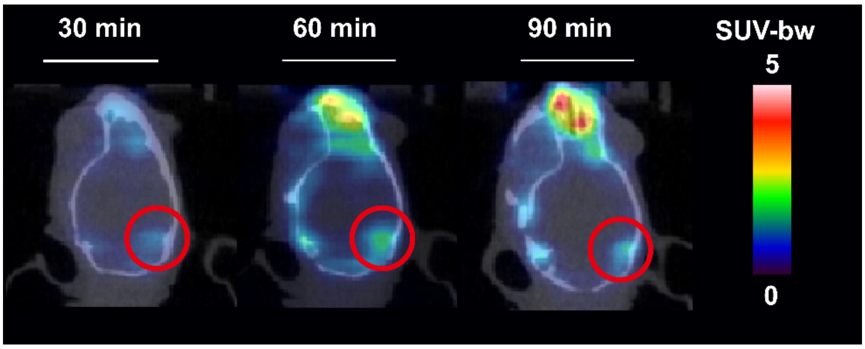

Figure S5. The microPET-CT coronal image of (2S,4S)4-[^{18}F]FPArg in HS683-Luc tumor-bearing nude mice at 30, 60 and 90 min point; where red circle indicates glioma area.

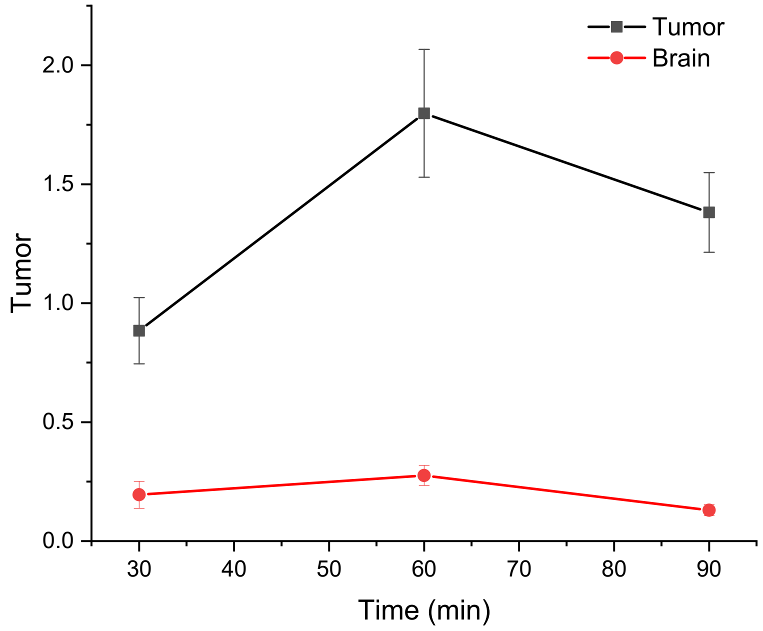

Figure S6. Time-activity curves of (2S,4S)4-[^{18}F]FPArg uptake in HS683-Luc tumor-bearing nude mice brain and tumor.

3. Estimated human dosimetry of (2S,4S)4-[^{18}F]FPArg.

Table S1. Estimated human dosimetry data of (2S,4S)4-[^{18}F]FPArg in mSv/MBq(male).

Target Organ	Total	EDE Cont.	ED Cont.
Adrenals	2.56E-03	0.00E+00	6.41E-06
Brain	5.39E-04	0.00E+00	1.35E-06
Breasts	1.80E-03	2.70E-04	9.00E-05
Gallbladder Wall	2.85E-03	1.71E-04	0.00E+00
LLI Wall	2.47E-03	0.00E+00	2.97E-04
Small Intestine	3.73E-03	2.24E-04	9.33E-06
Stomach Wall	2.41E-03	0.00E+00	2.89E-04
ULI Wall	2.73E-03	1.64E-04	6.82E-06
Heart Wall	2.00E-03	0.00E+00	0.00E+00
Kidneys	8.67E-03	5.20E-04	2.17E-04
Liver	4.09E-03	2.46E-04	2.05E-04
Lungs	2.40E-03	2.88E-04	2.88E-04
Muscle	1.46E-03	0.00E+00	3.65E-06
Ovaries	2.56E-03	6.41E-04	5.13E-04
Pancreas	2.64E-03	0.00E+00	6.60E-06
Red Marrow	1.93E-03	2.32E-04	2.32E-04
Osteogenic Cells	3.14E-03	9.41E-05	3.14E-05
Skin	1.50E-03	0.00E+00	1.50E-05
Spleen	1.85E-03	0.00E+00	4.62E-06
Testes	1.95E-03	0.00E+00	0.00E+00
Thymus	2.05E-03	0.00E+00	5.13E-06
Thyroid	1.96E-03	5.87E-05	9.78E-05
Urinary Bladder Wall	2.41E-03	0.00E+00	1.20E-04
Uterus	2.63E-03	0.00E+00	6.56E-06
Total Body	2.12E-03	0.00E+00	0.00E+00
Effective Dose Equivalent (mSv/MBq)		2.91E-03	
Effective Dose (mSv/MBq)			2.44E-03

4. NMR of compound **4**.

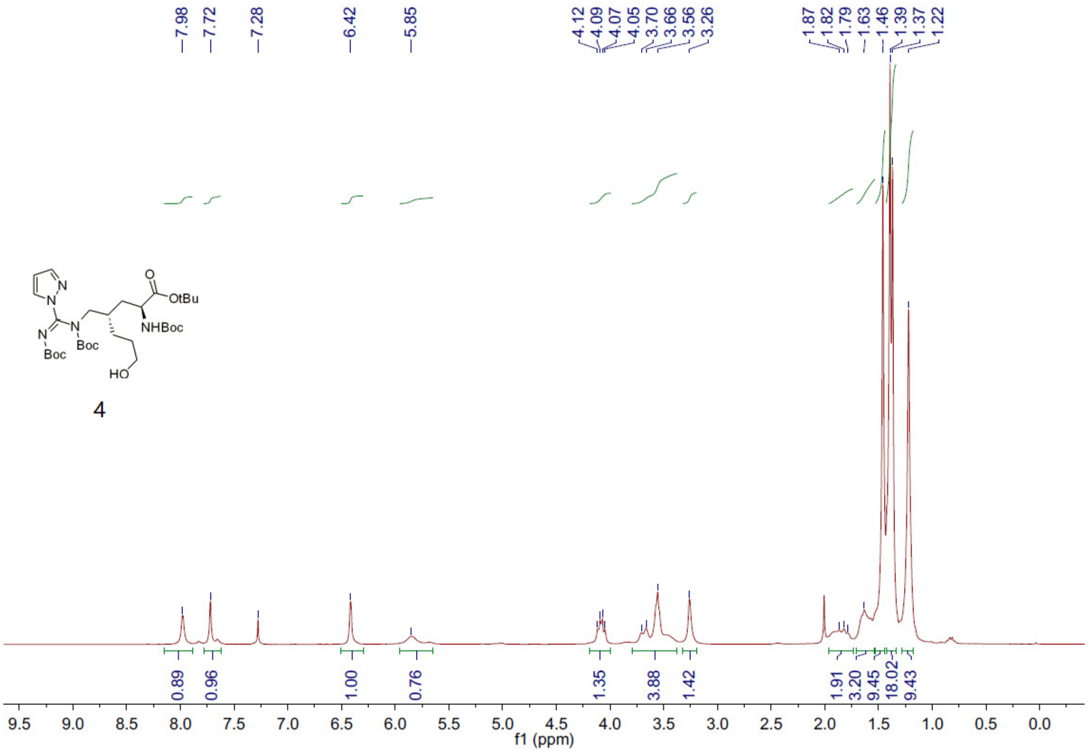

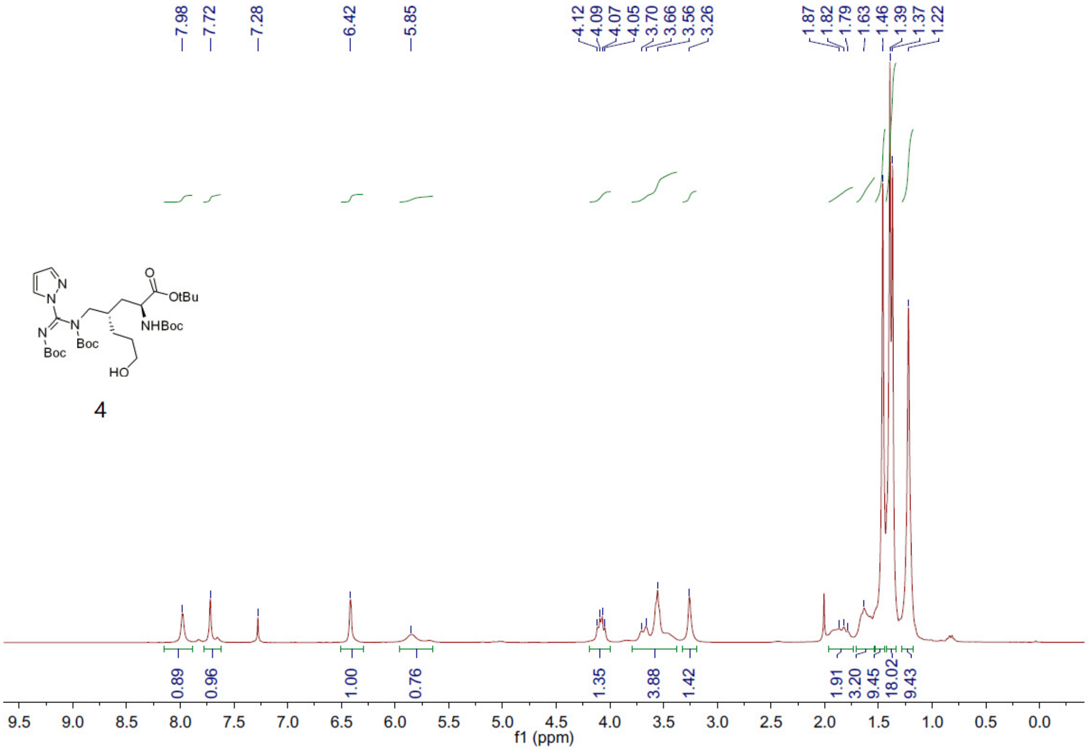

Supplement: Supplementary file 1 [file pharmaceuticals-15-00946-s001.zip › pharmaceuticals-1798706-supplementary.pdf]
